# A 10-Year Evaluation of Short-Term Outcomes After Synchronous Colorectal Cancer Surgery: a Dutch Population-Based Study

**DOI:** 10.1007/s11605-021-05036-8

**Published:** 2021-05-24

**Authors:** A. K. Warps, R. Detering, J. W. T. Dekker, R. A. E. M. Tollenaar, P. J. Tanis, on behalf of the Dutch ColoRectal Audit

**Affiliations:** 1 Department of Surgery, Leiden University Medical Center, Albinusdreef 2, 2333 ZA Leiden, The Netherlands; 2 Dutch Institute for Clinical Auditing, Rijnsburgerweg 10, 2333 AA Leiden, The Netherlands; 3 Department of Surgery, Amsterdam UMC, University of Amsterdam, Meibergdreef 9, 1105 AZ Amsterdam, The Netherlands; 4 Department of Surgery, Reinier de Graaf Groep, Reinier de Graafweg 5, 2625 AD Delft, The Netherlands; 5 Department of Surgery, Cancer Centre Amsterdam, Amsterdam UMC, University of Amsterdam, De Boelelaan 1117, 1081 HV Amsterdam, The Netherlands

**Keywords:** Synchronous colorectal cancer, Surgery, Time trend, Short-term outcomes, Tumor distribution

## Abstract

**Background:**

Synchronous colorectal cancer (CRC) has been associated with higher postoperative morbidity and mortality rates compared to solitary CRC. The influence of improved CRC care and introduction of screening on these outcomes remains unknown. This study aimed to evaluate time trends in incidence, population characteristics, and short-term outcomes of synchronous CRC patients at the population level over a 10-year time period.

**Methods:**

Data of all patients that underwent resection for primary CRC were extracted from the Dutch ColoRectal Audit (2010–2019). Analyses were stratified for solitary and synchronous colon and rectal cancer. Multilevel logistic regression analyses were used to determine factors associated with pathological and surgical outcomes.

**Results:**

Among 100,474 patients, 3.1% underwent surgery for synchronous CRC. A screening-related decrease for surgically treated left-sided solitary and synchronous colon cancer and a temporary increase for exclusively right-sided colon cancer were observed. Synchronous CRC patients had higher rates of complicated postoperative course, failure to rescue, and mortality. Bilateral synchronous colon cancer was more often treated with subtotal colectomy (25.4%) and demonstrated higher rates of surgical complications, reinterventions, prolonged hospital stay, and mortality than other synchronous tumor locations.

**Discussion:**

National bowel screening resulted in contradictory effects on surgical resections for synchronous CRCs depending on sidedness. Bilateral synchronous colon cancer required more often extended resection resulting in significantly worse outcomes than other synchronous tumor locations. Identification of low volume, high complex CRC subpopulations is relevant for individualized care and has implications for case-mix correction and benchmarking in clinical auditing.

**Supplementary Information:**

The online version contains supplementary material available at 10.1007/s11605-021-05036-8.

## Introduction

Synchronous colorectal cancer (CRC) is defined as the occurrence of more than one colorectal tumor at the same time or within 6 months of the initial diagnosis.^1^ The reported incidence of synchronous CRC ranges from 1.1 to 10.7%.^2^–^9^ Previous studies have demonstrated that these tumors are associated with advanced age, history of inflammatory bowel disease, or colorectal adenoma^2^, ^5^, ^7^, ^10^, ^11^ and are commonly located in the proximal colon.^2^, ^5^, ^7^, ^10^

In 2014, the national bowel cancer screening was introduced in the Netherlands, which has resulted in a shift towards more early-stage CRC diagnosis.^12^ Studies have indicated that this has led to a more extensive preoperative workup,^13^–^15^ a different surgical approach,^16^, ^17^ and lower postoperative morbidity and mortality rate.^12^ These studies often excluded synchronous CRC, while especially those patients might benefit from detection at an early stage, thereby potentially limiting the need for more extensive surgery.^7^, ^9^, ^11^, ^18^ Although surgical resection of CRC remains the gold standard for curative treatment, the required extent of the procedure for tumors located in multiple segments has remained controversial in the past decades.^19^–^23^ This is mainly, since patients with synchronous tumors often require an extensive resection, because they are more likely to develop local tumor recurrence within three years.^4^, ^8^, ^24^ However, more extensive resections are accompanied by higher postoperative complication rates,^18^, ^21^ and the impact on long-term survival is unclear.^4^, ^5^, ^7^–^9^, ^24^, ^25^

The Dutch ColoRectal Audit (DCRA) is a nationwide registry with high completeness and validity,^26^ which collects data on patient, tumor, and surgical characteristics of all patients who undergo resection for primary CRC, including patients with synchronous CRC. A previous study of the DCRA in 2011 demonstrated that patients with synchronous CRC had more often an advanced tumor stage, required more extended surgical procedures, and resulted in higher postoperative morbidity, reintervention, and mortality rate compared to solitary CRC patients.^18^ This study was performed before the introduction of the FIT screening, and no stratification for synchronous colon and rectal cancer was used. Furthermore, several other changes in CRC management have occurred with an overall improvement in short-term outcomes.^27^ We hypothesized that synchronous CRC is decreasing in incidence related to screening, that this subpopulation might also have benefitted from overall improvements in CRC care, but that also heterogeneity within the synchronous CRC population might exist with some patients requiring specific attention to optimize outcomes.

The aim of the present study was primarily to determine time trends in incidence, population, and treatment characteristics and short-term outcomes (incomplete resection margin, complicated course, failure to rescue, mortality) of surgically treated synchronous CRC patients based on a nationwide clinical audit during a 10-year time period.

## Methods

This study comprises a population-based observational cohort study with data derived from the DCRA. This disease-specific national audit collects information on patient, tumor, treatment, and short-term outcome characteristics of all patients undergoing resection for primary CRC in the Netherlands.^26^ No ethical approval or informed consent was required, as stated by the Dutch law.

### Study Population and Subpopulations

All patients who underwent surgery for a first primary colon or rectal carcinoma between January 1^st^, 2010, and December 31^th^, 2019, were potentially eligible for this study. The study population was divided into four main subgroups: solitary colon cancer, synchronous colon cancer, solitary rectal cancer, and synchronous rectal cancer. If one of the synchronous tumors was located in the rectum, the patient was assigned to the rectal cancer study population due to the higher complexity of rectal cancer surgery. Synchronous colorectal tumors were divided into subgroups based on the tumor location: right-right colon, left-left colon, right-left colon, right colon-rectum, left colon-rectum, and rectum-rectum.

### Data Extraction, Outcome Parameters, and Definitions

The following data were extracted from the DCRA-database: patient and disease characteristics, procedural characteristics, and postoperative outcomes within 30 days after resection or in-hospital events. The 30-day postoperative outcomes were the overall non-surgical complication rate (including pulmonary, cardiac, thromboembolic, infectious, and neurological complications), surgical complication rate (including anastomotic leakage, ileus, abscess, fascial dehiscence, bowel perforation, ureter/bladder perforation, and wound infection), reintervention rate for a surgical complication (consisting of endoscopic, image-guided, and surgical reinterventions), and readmission rate within 30 days after surgery. Combined surgical and non-surgical complications were analyzed in the group with surgical complication. Complications, reinterventions, and readmissions (registered since 2012) were recorded for the first 30 days after resection until 2018. Since then, the 90-day outcomes are registered.

In addition, three 30-day outcome indicators of the DCRA were assessed, including complicated course, mortality, and failure to rescue**.** A complicated postoperative course is defined in the DCRA as any complication resulting in a length of hospital stay of more than 14 days, or a surgical complication requiring reintervention or death within 30 days after surgery during the primary hospital visit. Postoperative mortality is defined as death within the first 30 days after resection or during index hospital admission (including patients with and without a complicated course). Failure to rescue is defined as the failure rate to prevent mortality after the occurrence of a complication after elective CRC surgery and is calculated by dividing the number of patients who died after a complicated postoperative course by the total number of patients with a complicated postoperative course. Failure to rescue is considered a team effort in CRC surgery.^28^, ^29^ For this reason, CRC was analyzed as one group. For pathological outcomes, the tumor-free resection margin rate was evaluated, defined as a tumor-free bowel resection margin as well as a tumor-free retroperitoneal resection margin for colon cancer and negative circumferential resection margin for rectal cancer (> 1 mm).

### Statistical Analyses

Postoperative surgical and pathological outcomes were evaluated for solitary and synchronous colon and rectal cancer. Categorical or dichotomous outcomes are reported as absolute numbers with percentages and were compared using the Pearson chi-square test. To evaluate the time trend in CRC resections, the absolute numbers of CRC resections per tumor location were calculated for each year.

Among patients with synchronous CRC, type of surgical procedure and postoperative complication rate was analyzed for each of the predefined tumor locations. Time trends for incomplete resection margin rates, complicated course, failure to rescue, and mortality after colon and rectal cancer surgery were analyzed for each year. Analyses of resection margin were stratified for T1–3 and T4 stage, in which the patient was assigned to the T4 group if at least one of the tumors was classified as T4. Differences in these outcomes between the registration years 2010 and 2019 were assessed by using a Pearson chi-square test.

Multilevel logistic regression analyses were used to assess factors associated with the previously described postoperative outcomes. Multilevel logistic regression analyses provide a more accurate estimate when dealing with hierarchically structured data than traditional multivariable logistic regression analyses as it accounts for a dependency of patients within hospitals.^30^, ^31^ Complicated course, failure to rescue, and mortality were corrected for the case-mix variables of the DCRA^26^, ^32^: sex, body mass index (BMI), age, Charlson Comorbidity Index (CCI), American Society of Anesthesiologists (ASA) score, preoperative tumor complications (e.g., anemia, perforation, obstruction/ileus or peri-tumoral abscess), elective or emergency resection, additional resection due to metastasis or tumor ingrowth, T-stage, M-stage, and neoadjuvant radiotherapy for rectal cancer.

The incomplete resection margin rate was corrected for preoperative tumor complications, setting (emergency, elective), T-stage, N-stage, M-stage, and synchronous tumors. For rectal cancer, neoadjuvant radiotherapy was added to the model. T-stage and N-stage were based on the pathological tumor stage for colon cancer, and clinical tumor stage for rectal cancer, because of the differences in the reliability of clinical staging based on imaging (CT for colon cancer and MRI for rectal cancer) and the application of down-staging treatment (seldom in colon cancer and regularly in rectal cancer).

Multicollinearity was assessed with the variance inflation factor (VIF). A VIF >2.5 was considered multicollinear leading to the removal of one of the variables. Results are reported in adjusted odds ratio (AOR) and 95% confidence interval (CI). Statistical significance was defined as p-value <0.05. RStudio version 1.3.959 (2020) was used for statistical analyses.

## Results

**Fig. 1:**
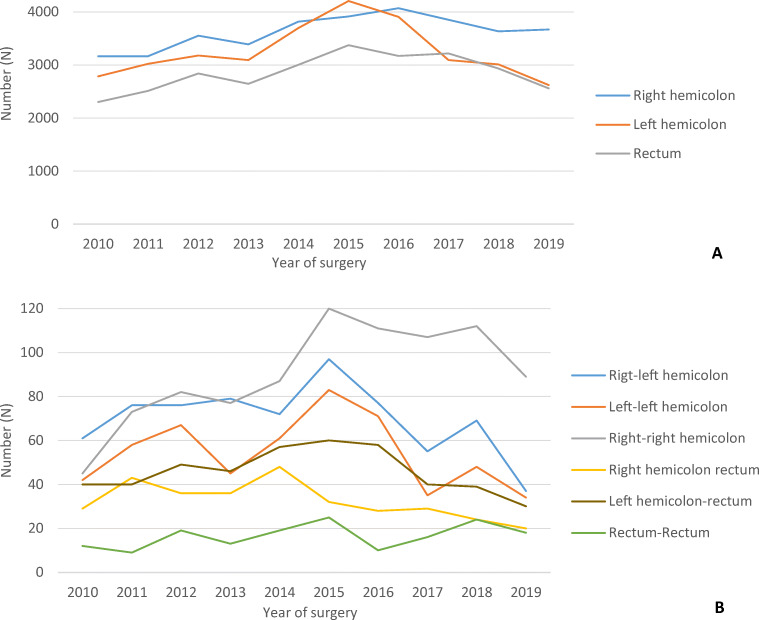
**A** Time trend of the number of oncologic resections between 2010 and 2019 for solitary tumors located in the right-hemicolon, left-hemicolon, and rectum. **B** Time trend of the number of oncologic resections between 2010 and 2019 for synchronous tumors located in right-left hemicolon, left-left hemicolon, right-right-hemicolon, right-hemicolon-rectum, left-hemicolon-rectum, and rectum-rectum

Of the total 100,474 CRC patients, 3.1% (*n*=3095) underwent surgery for synchronous CRC with the majority of the surgical treated synchronous CRC both being located in the right colon (29.1%). The total colon cancer study population consisted of 70,979 patients, with 2146 (3.0%) having a synchronous colon tumor, whereas 29,495 patients had rectal cancer with 949 (4.4%) patients having a synchronous tumor (at least one of both tumors located in the rectum). The majority of the synchronous cancers were both located in the right colon (29.2%), followed by right-left colon (22.6%), left-left colon (17.6%), left colon-rectum (14.8%), right colon-rectum (10.5%), and rectum-rectum (5.3%) (Table 1). Since 2015 (1 year after the introduction of bowel cancer screening in the Netherlands), the number of oncological resections for both solitary (Fig. 1A) and synchronous (Fig. 1B) left-sided colon cancer and rectal cancer showed a substantial decrease, whereas this was much later seen and to a lesser extent for right-sided solitary colon cancer, and with a temporary increase for right-right synchronous cancers.

**Table 1:** Baseline characteristics of the solitary and synchronous colorectal cancer study population

		Colon cancer study population			Rectal cancer study population		
		Solitary *N*=68,833	Synchronous *N* = 2146	p-value	Solitary *N*=28,546	Synchronous^A^ *N*=949	p-value
Patient characteristics							
Age	<60	11,676 (17.0)	227 (10.6)	<0.001	7203 (25.2)	132 (13.9)	<0.001
	60–70	20,930 (30.4)	566 (26.4)		9832 (34.4)	306 (32.2)	
	70–80	23,813 (34.6)	845 (39.4)		8529 (29.9)	359 (37.8)	
	>80	12,407 (18.0)	508 (23.7)		2,977 (10.4)	152 (16.0)	
	Missing	7	0		5	0	
Sex	Male	36,122 (52.5)	1162 (54.1)	0.135	17,929 (62.8)	694 (73.1)	<0.001
	Female	32,697 (47.5)	984 (45.9)		10,607 (37.2)	255 (26.9)	
	Missing	14	0		10	0	
BMI	<18.5	1205 (1.8)	43 (2.1)	0.246	437 (1.6)	10 (1.1)	0.449
	18.5–25.0	26,290 (39.9)	781 (37.9)		11,047 (39.8)	361 (39.0)	
	25.0–30.0	25,803 (39.1)	840 (40.8)		11,395 (41.0)	378 (40.9)	
	≥30	12,059 (18.3)	384 (18.6)		4728 (17.0)	171 (18.5)	
	Missing	575	13		168	5	
ASA	I-II	49,877 (72.5)	1449 (67.5)	<0.001	23,159 (81.1)	709 (74.7)	<0.001
	III+	18,856 (27.4)	695 (32.4)		5355 (18.8)	240 (25.3)	
	Missing	100	2		32	0	
CCI	0-I	49,849 (72.4)	1494 (69.6)	0.005	22,167 (87.7)	669 (70.5)	<0.001
	II+	18,984 (27.6)	652 (30.4)		6379 (22.3)	280 (29.5)	
Preoperative characteristics							
Tumor complication^B^	No	43,360 (63.0)	1309 (61.0)	0.047	23,149 (81.1)	701 (73.9)	<0.001
	Yes	25,234 (36.7)	834 (38.9)		5296 (18.6)	247 (26.0)	
	Missing	239	3		101	1	
Neoadj radiotherapy	No	68,328 (99.3)	2136 (99.5)	0.110	10,389 (36.5)	508 (53.5)	<0.001
	Yes	346 (0.5)	5 (0.2)		18,124 (63.5)	440 (46.4)	
	Missing	159 (0.2)	5 (0.2)		33 (0.1)	1 (0.1)	
cT stage	cT1-2	9156 (13.3)	278 (13.0)	<0.001	8626 (30.2)	279 (29.4)	<0.001
	cT3	13,294 (19.3)	462 (21.5)		15,396 (53.9)	503 (53.0)	
	cT4	3117 (4.5)	140 (6.5)		2666 (9.3)	67 (7.1)	
	cTx	43,266 (62.9)	1266 (59.0)		1858 (6.5)	100 (10.5)	
cN stage	cN0	21,616 (31.4)	635 (29.6)	0.009	12,853 (45.0)	392 (41.3)	<0.001
	cN1-2	9847 (14.3)	355 (16.5)		13,854 (48.5)	387 (40.8)	
	cNx	37,370 (54.3)	1156 (53.9)		1839 (6.4)	170 (17.9)	
Site	Right- (right)-colon^c^	36,224 (52.6)	903 (42.1)	<0.001	-	-	<0.001
	Left- (left)- colon^c^	32,609 (47.4)	544 (25.3)		-	-	
	Right-left colon	-	699 (32.6)		-	-	
	Right colon-rectum	-	-		-	325 (34.2)	
	Left colon-rectum	-	-		-	459 (48.4)	
	Rectum- (rectum)^C^	-	-		28,546 (100.0)	165 (17.4)	
Operative characteristics							
Setting	Elective	58,291 (84.7)	1913 (89.1)	<0.001	28,085 (98.4)	912 (96.1)	<0.001
	Emergency	10,476 (15.2)	232 (10.8)		427 (1.5)	34 (3.6)	
	Missing	66	1		34	3	
Technique	Open	23,887 (34.7)	930 (43.3)	<0.001	7306 (25.6)	347 (36.6)	<0.001
	Laparoscopic^D^	44,231 (64.3)	1199 (55.9)		19,274 (67.5)	579 (61.0)	
	Local excision	61 (0.1)	2 (0.1)		1707 (6.0)	18 (1.9)	
	Missing	654	15		259	5	
Anastom.	Anastom.	59,710 (86.7)	1731 (80.7)	<0.001	7860 (27.5)	287 (30.2)	0.001
	Anastom. + stoma	1974 (2.9)	108 (5.0)		7771 (27.2)	215 (22.7)	
	Stoma	6730 (9.8)	301 (14.0)		11,320 (39.7)	427 (45.0)	
	Missing	66	1		1595	20	
Additional resection for local ingrowth	No	61,211 (88.9)	1922 (89.6)	1.000	24,648 (98.4)	842 (88.7)	0.227
	Yes	6460 (9.5)	203 (9.6)		1932 (6.8)	77 (8.1)	
	Missing	179	21		1966	30	
Additional resection for metastases	No	66,235 (96.5)	2067 (96.5)	1.000	26,203 (91.8)	896 (94.4)	0.096
	Yes	2419 (3.5)	75 (3.5)		776 (2.7)	36 (3.8)	
	Missing	94	4		1567	17	

^A^Synchronous rectal cancer and rectum-colon cancer

^B^Tumor complications includes anemia, perforation, obstruction/ileus, or peri-tumoral abscess

^C^In the case of synchronous tumors, both tumors are located at the same site

^D^Laparoscopy included conventional laparoscopic procedures, robot-assisted laparoscopic procedures, and transanal total mesorectal excision (TaTME, rectal cancer)

Neoadj radiotherapy, neoadjuvant radiotherapy; Anastom., anastomoses. Missing values of less than 15% are only shown as numbers

### Solitary vs. Synchronous Colorectal Cancer

Compared to solitary CRC, patients with synchronous colon and rectal tumors were older and had more often an ASA score III+, a CCI II+, and tumor-related complications as shown in Table 1. A male predominance was found if one of both tumors was located in the rectum compared to a solitary rectal cancer (73.1 to 62.8%, p<0.001). 

A more advanced T and N stage was seen in patients with synchronous tumors compared to solitary tumors, as displayed in Table 2. Regarding treatment characteristics, it was found that synchronous rectal cancer patients underwent less frequent neoadjuvant radiotherapy (46.4% to 63.5%, p<0.001), with the least often for right colon-rectum (39.7%), followed by left colon-rectum (46.3%) and rectum-rectum tumors (60.0%). Synchronous rectal resection was more often performed in the emergency setting compared to solitary rectal cancer (3.6% vs. 1.5%, p<0.001), which was in contrast to synchronous colon tumors (10.8% vs. 15.2%, p<0.001). For both synchronous cancer populations, there was a higher rate of open resection (43.3% vs. 34.7%, p<0.001 (colon); 36.6% vs. 25.6%, p<0.001 (rectum)) and stoma creation without primary anastomosis (14.0% vs. 9.8%, p<0.001 (colon); 45.0% vs. 39.7% p=0.001 (rectum)).

**Table 2:** Pathologic characteristics and postoperative outcomes of solitary and synchronous colorectal cancer

		Colon cancer study population			Rectal cancer study population		
		Solitary *N*=68,833	Synchronous *N*= 2146	p-value	Solitary *N*=28,546	Synchronous^A^ *N*=949	p-value
Pathology							
(y)pT-stage	(y)pT0-1	6591 (9.6)	103 (4.8)	<0.001	5512 (19.3)	77 (8.1)	<0.001
	(y)pT2	11,606 (16.9)	321 (15.0)		8710 (30.5)	251 (26.4)	
	(y)pT3	38,335 (55.7)	1320 (61.5)		12,412 (43.5)	532 (56.1)	
	(y)pT4	11,417 (16.6)	378 (17.6)		1319 (4.6)	85 (9.0)	
	(y)pTX	884	24		593	4	
(y)pN-stage	(y)pN0	40,363 (58.6)	1169 (54.5)	<0.001	17,570 (61.5)	550 (58.0)	<0.001
	(y)pN1-2	27,575 (40.1)	950 (44.3)		9368 (32.9)	373 (39.3)	
	(y)pNx	895	236		1608	26	
M-stage	M-	60,896 (88.5)	1910 (89.0)	0.466	26,279 (92.1)	845 (89.0)	0.001
	M1	7937 (11.5)	236 (11.0)		2267 (7.9)	104 (11.0)	
Resection margin	Tumor-free	66,119 (96.1)	2067 (96.3)	0.120	25,735 (90.2)	882 (92.9)	0.970
	Incomplete	1574 (2.3)	61 (2.9)		1813 (6.4)	63 (6.6)	
	Missing	1140	18		998	4	
Number of lymph nodes	<12	13,864 (20.1)	221 (10.3)	<0.001	9039 (31.7)	202 (21.3)	<0.001
	≥ 12	54,543 (79.2)	1905 (88.8)		17,872 (62.6)	726 (76.8)	
	Missing	426	20		1635	21	
Positive lymph nodes	No	40,843 (59.3)	1144 (53.3)	<0.001	17,670 (61.9)	529 (55.7)	<0.001
	Yes	27,196 (39.5)	975 (45.4)		9099 (31.8)	393 (41.4)	
	Missing	794	27		1777	27	
Postoperative outcomes							
Complications	No	48,908 (71.1)	1279 (59.6)	<0.001	18,462 (64.7)	555 (58.5)	<0.001
	Non-surgical	9043 (13.1)	386 (18.0)		4073 (14.3)	159 (16.8)	
	Surgical	10,822 (15.8)	481 (22.4)		6011 (21.1)	235 (24.8)	
Reintervention	No	62,916 (91.4)	1865 (86.9)	<0.001	25,134 (88.0)	813 (85.7)	0.030
	Yes	5917 (8.6)	281 (13.1)		3412 (12.0)	136 (14.3)	
LOS > 14 days	No	58,851 (85.5)	1632 (76.0)	<0.001	23,431 (82.1)	726 (76.5)	<0.001
	Yes	9623 (14.0)	504 (23.5)		4920 (17.2)	218 (23.0)	
	Missing	359	10		195	5	
Readmission^B^	No	52,419/56,668 (92.5)	1612/1791 (90.0)	<0.001	20,466/23,734 (86.2)	674/776 (86.9)	0.688
	Yes	4140/56,668 (7.5)	175/1,791 (10.0)		3222/23,734 (13.8)	101/776 (13.0)	
	Missing	129	4		40	1	
Complicated course	No	57,827 (84.0)	1593 (74.2)	<0.001	22,815 (79.9)	703 (74.1)	<0.001
	Yes	11,006 (16.0)	553 (25.8)		5731 (20.1)	246 (25.9)	
Failure to rescue^c^	No	12,039/13,429 (80.6)	596/685 (87.0)	0.033			
	Yes	1390/13,429 (10.4)	89/685 (13.0)				
Mortality	Survival	66,731 (96.9)	2051 (95.6)	<0.001	28,100 (98.4)	913 (96.2)	<0.001
	Mortality	2102 (3.1)	95 (4.4)		446 (1.6)	36 (3.8)	

Postoperative outcomes after primary solitary and synchronous colorectal cancer resection. Missing values of less than 15% are only shown as numbers

^A^If one of the synchronous tumors was located in the rectum, the patient was assigned to the synchronous rectal cancer group

^B^Readmission was registered since 2012

^C^The percentage of patients with failure to rescue is analyzed for the total number of elective colorectal cancer resections with a complicated course

### Type of Surgery for Synchronous Cancers

**Fig. 2:**
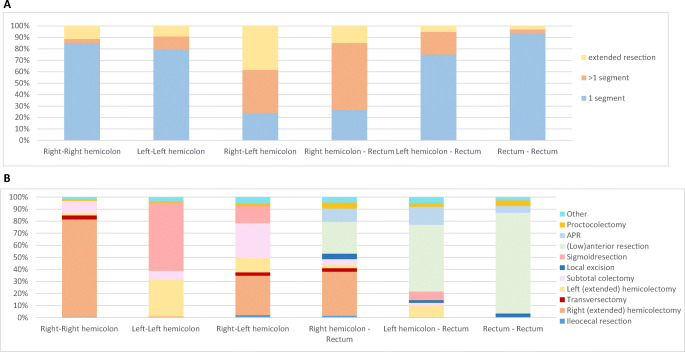
**A** Type surgical procedure (1 segmental resection, >1 segmental resection, extended resection (including subtotal colectomy and proctocolectomy) stratified for synchronous colorectal cancer location. **B** Surgical procedures performed stratified for synchronous colorectal cancer resection. Percentages of each type of resection are based on the total number of resections performed

Bilateral colon tumors and synchronous colon-rectum tumors often required resection of multiple segments (right-left colon 37.6%; right colon-rectum 58.8%; left colon-rectum 19.8%). When the right colon was involved, an extended resection was most often performed in case of bilateral location (38.3%) (Fig. 2A), with the most commonly performed procedures being a right (extended) hemicolectomy, sigmoid resection, and (low) anterior resection (Fig. 2B). Extended resections were most often performed for bilateral located colon cancer (38.3%), consisting of a subtotal colectomy in 25.4%, and a proctocolectomy in 4.5%. The extent of resection was comparable in the elective and emergency setting (one segment in 63.4% vs. 63.8%, multiple segments in 15.5% vs. 16.8%, and (sub)total colectomy in 21.1% vs. 19.4%, p=0.765).

### Short-Term Outcomes

**Fig. 3:**
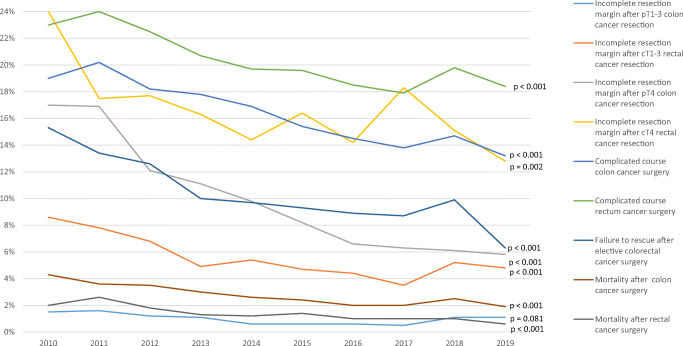
Time trend of the primary short outcomes after colorectal cancer resection. A chi-square test was used for calculating the p-values for 2010 vs. 2019

**Fig. 4:**
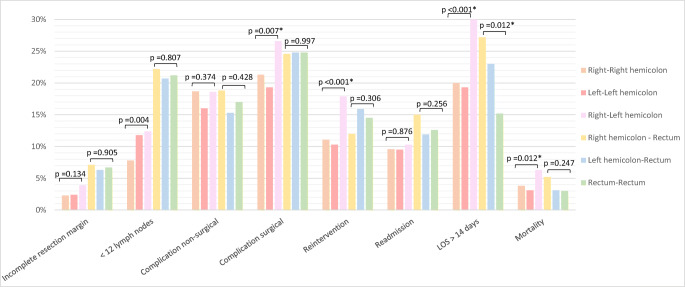
Short-term postoperative outcomes after primary synchronous colorectal cancer resection per tumor location. The readmission rate is calculated for 2012–2019. A chi-square test was used to calculate the p-values

Time trend analyses of the 30-day surgical outcomes and pathological outcomes after colorectal cancer surgery revealed improvement during the study period as shown in Fig. 3. When compared to solitary cancer, both patients with synchronous colon cancer as well as rectal cancer demonstrated significantly higher rates of non-surgical complications, surgical complications, reinterventions, complicated course, and failure to rescue (Table 2). The primary admission of synchronous CRC patient was more often longer than 14 days. In contrast to the rectal cancer study population, patients with synchronous colon cancer were more frequently readmitted within 30 days after primary surgery than patients with solitary tumors (10.0% vs. 7.5%, p<0.001). Concerning pathological outcomes, significantly higher proportions of incomplete resection margin were found for both T1–3 and T4 stage synchronous colon cancers (3.1% vs. 0.9%, p<0.001 and 13.5% vs. 9.5%, p=0.009). Synchronous rectal cancer resection showed more often a lymph node harvest of 12+ than solitary cancer (76.8% vs. 62.6%, p<0.001). Subgroup analysis of short-term outcomes for synchronous colon cancer showed that tumor location in both the right and left colon, if compared to right-right and left-left tumor locations, had significantly more surgical complications (26.6% vs. 21.3% and 19.3%; p=0.007), reinterventions (17.9% vs. 11.1% and 10.3%; p<0.001), and higher mortality rate (6.3% vs. 3.8% and 3.1%; p=0.012). The proportion of patients with a primary hospital stay of more than 14 days was significantly higher in both synchronous colon cancer (31.2% (right-left) vs. 20.0% (right-right) vs. 19.3% (left-left); p<0.001) and synchronous rectal cancer (27.2% right colon-rectum) vs. 23.0% (left colon-rectum) and 15.2% (rectum-rectum); p=0.012) (Fig. 4).

Multilevel logistic regression analyses (Suppl. Table 1) showed that synchronous colon cancer was independently associated with an increased odds for incomplete resection margin for both T1–3 stage (AOR 3.848, 95% CI 2.896–5.114) and T4 stage (AOR 1.676, 95% CI 1.238–2.269), but this was not observed if one of the tumors was located in the rectum. Synchronous colon as well as rectal tumors revealed a significantly higher odds for complicated course (AOR 1.796, 95% CI 1.614–1.998 and AOR 1.265, 95% CI 1.072–1.491) and mortality (AOR 1.361, 95% CI 1.061–1.747 and AOR 1.924, 95% CI 1.262–2.932), but not for failure to rescue (both synchronous colon or rectal cancer) (Suppl. Table 2).

## Discussion

This population-based study evaluated 10 years of surgery for synchronous CRC in the Netherlands. In-depth analyses of time trends in incidence as well as evaluation of different subgroups depending on sidedness of the multiple cancers are provided. Overall, 3.1% of the patients underwent surgery for synchronous CRC, with both tumors most frequently located in the right colon. Although outcomes of CRC care improved over time, patients with synchronous tumors still had higher rates of incomplete resection margin, complicated course, failure to rescue, and mortality if compared to patients with a solitary tumor. Subgroup analyses of synchronous tumors based on tumor location revealed that bilateral synchronous cancers most often underwent subtotal colectomy and proctocolectomy and had the worst outcome compared to left-left and right-right colon cancer locations. This underlines the importance of identifying high-risk patient groups that need to be optimally informed by treating physicians and also require individualized patient care to improve outcomes. In addition, our results also highlight the need for continuous evaluation of delivered care over time because benchmarks are subject to change.

Previous studies have demonstrated controversial results for the distribution of synchronous CRC. A study of van Leersum et al. showed that synchronous tumors had a predominant bilateral location,^18^ whereas Mulder et al. found that most often synchronous cancers were both in the right colon.^5^ This is in line with our results that showed alternating highest resection numbers for right-right and right-left colon cancer sidedness before 2014, with a predominant right-right distribution since 2014. This is most likely explained by the introduction of the fecal immunochemical test (FIT) as part of the bowel cancer screening program in 2014. Several studies have hypothesized that serrated lesions are more often located in the right colon and that these lesions are less likely to bleed compared to the flat shape of adenomas.^33^–^35^ As a consequence, studies found that CRC screening tests detect right-sided tumors less often and in a more advanced stage than left-sided colon and rectal tumors.^13^, ^36^, ^37^ Although we included only patients who were surgically treated, the results are in line with these previous studies.

A study by de Neree et al. showed that screening-detected cancer is more often diagnosed at an early stage, resulting in less extensive surgical procedures with less postoperative complications and mortality.^12^ Despite the fact that more early-stage CRC is detected, synchronous colon tumors were still diagnosed in a more advanced stage compared to solitary carcinomas. Although our results showed a decrease in complicated course, failure to rescue, and mortality over a 10-year time period, the rate was still higher for synchronous cancer patients compared to solitary cancer patients. We suggest that this is caused by the more complex surgical procedure that is generally required for synchronous cancer, illustrated by a more frequent open approach and the creation of a stoma without anastomosis. We also found that patients with at least one of the cancers located in the rectum had almost a similar clinical tumor stage as patients with solitary rectal cancer. Remarkably, they received less often neoadjuvant radiotherapy but had a higher complication rate. This suggests that the surgical procedures required for synchronous rectal cancers determine the morbidity rate, and not the neoadjuvant treatment. In addition, the presence of a synchronous tumor was independently associated with worse short-term outcomes, and in colon cancer also with incomplete resection margin. Most studies and clinical audits exclude patients with synchronous CRC, partially due to a small number of synchronous CRC with different features that result in worse short-term outcomes compared to solitary CRC patients. The findings of the present study have implications for benchmarking and case-mix adjustment in clinical auditing. Surgically treated synchronous CRC patients need to be incorporated into the overall evaluation of CRC care, and synchronous CRC should then be added as a variable to the case-mix model.

Over the past decades, there has been an ongoing discussion to which extent the surgical procedure needs to be performed for CRC located in multiple segments.^19^–^23^ A more extended surgical procedure has been associated with a higher postoperative morbidity and mortality rate^18^, ^21^ but a lower recurrence rate, whereas multiple segmental resections have been associated with more recurrences but less postoperative morbidity.^8^, ^21^, ^24^ You et al. showed that (sub)total colectomy was more commonly performed for multiple polyps or malignancies, with a higher incidence of ileus/small bowel obstructions and overall complications as well as a longer hospital stay. These patients experienced a lower quality of life compared to patients that underwent a segmental resection due to a lower median number of stools per day.^38^ Bakker et al. evaluated 15,667 patients who underwent resection for colon cancer and found that a subtotal colectomy was associated with an increased risk for anastomotic leakage but not with subsequent death.^39^ Nevertheless, Kilma et al. found a 2.4 fold increase in the risk of mortality by a subtotal colectomy.^40^ Thiels et al. suggest that segmental resection should be performed whenever possible because there is no benefit in survival of (sub)total colectomy.^41^ Interestingly, Holubar et al. found that creating two non-diverting colonic anastomoses was associated with lower anastomotic leakage, intra-abdominal abscess, and surgical site infection rates compared to a single extended resection.^20^ Our results demonstrate that patients with synchronous bilateral colon cancer and right colon-rectum cancer more often underwent multiple segment resections and extended resections, with a subtotal colectomy or proctocolectomy more often performed in bilateral colon cancer than other synchronous colon cancer distributions. These patients demonstrated the highest postoperative complication, reintervention, and mortality rate compared to other synchronous colon tumor distributions, which is in line with the results of Lee et al..^42^

Although the association between synchronous CRC and long-term survival remains unclear,^4^, ^5^, ^7^–^9^, ^24^, ^25^ it is well known that postoperative complications, especially infectious complications, are associated with an increase in local recurrence rate and a decrease in overall survival.^43^ Besides, studies show that the local recurrence rate is higher for synchronous cancer than for solitary cancer.^4^, ^8^, ^24^ Even though long-term outcomes are not available in the DCRA, our findings support the conclusion of a study by Cecchini et al., which stated that segmental or regional colonic resections were appropriate in the elective setting based on their results of short- and long-term outcomes and that the indication for a (sub)total colectomy should not be based on the oncological outcomes.^23^ A potential strategy to minimize the need for an extended resection and to improve long-term survival is to detect synchronous CRC at an early disease stage that might allow for endoscopic or surgical local excisions. For this reason, a colonoscopy of the entire colon to detect synchronous tumors is essential. A synchronous tumor can easily be missed, due to obstruction, poor bowel preparation, or physical status of the patient resulting in incomplete preoperative colonoscopy.^44^ In case of incomplete visualization of the colon, it has been suggested that the detection rate of a synchronous tumor might be improvement by is an intra-operative colonoscopy.^45^ Sasaki et al. demonstrated that this resulted in detection of a synchronous lesion in 26.8% and a synchronous CRC in 4% of patients with a left-sided CRC, with a change in surgical procedure in 8.9%.^46^ We suggest that patient selection for extended resection is important in reducing adverse outcomes and mortality, taking the risk of disease recurrence into account. However, the right patient selection for a segmental or extended resection is not only based on objective clinical parameters, but also requires a well-informed patient who is able to make an informed choice whether the treatment option with its risks and benefits is the best considering their preferences and condition.^47^, ^48^ An individualized patient approach and shared decision-making are essential in patients with synchronous CRC to achieve good short-term and long-term outcomes. However, no definitive recommendations can be made based on the present study, due to the lack of information regarding long-term survival and disease recurrence.

The following limitations of this population-based study should be mentioned. The DCRA only registers short-term outcomes, and for this reason, the association between synchronous tumors and long-term oncological outcomes could not be evaluated. Due to the continuous development of the DCRA, parameters have been changed. Until 2017 the 30-day, complication rate, reintervention rate, and readmission rate were registered, and since 2018, the 90-day outcome rates were registered. This resulted in a slight overestimation of the 30-day outcomes in 2018 and 2019. In the DCRA, no distinction is made between hemicolectomy and extended hemicolectomy, which might have been relevant for comparing synchronous and solitary cancer.

## Conclusion

In this 10-year population-based study, 3.1% of the resection was performed for synchronous CRC, with most synchronous tumors both located on the right side. The introduction of the bowel cancer screening program has resulted in a decrease of resections for left-sided and rectal solitary and synchronous cancers, in contrast to a remarkable temporary increase in exclusively right-sided synchronous cancer. Due to the more advanced tumor stage and common involvement of multiple segments in synchronous CRC, more complex surgery is required, resulting in a higher postoperative complication and mortality rate and a longer stay in hospital compared to solitary tumors. Efforts should be made for detecting synchronous CRC at an early stage to reduce the need of extended resections. Patient selection and shared decision-making for extended resections are essential in the treatment of synchronous CRC. Besides, it is important to monitor and compare the results of synchronous CRC resections between hospitals for the improvement of quality of surgical care.

## Supplementary Information

ESM 1 (DOCX 24 kb).

## Data Availability

The data that support the results of the present study are available from the Dutch Institute for Clinical Auditing (DICA) but are not publicly available. Data are however available from the authors upon reasonable request and with permission of the Dutch Institute for Clinical Auditing and the Dutch ColoRectal Audit Board.
