# Supplementary material for: A 10-Year Evaluation of Short-Term Outcomes After Synchronous Colorectal Cancer Surgery: a Dutch Population-Based Study
Source: J Gastrointest Surg. 2021 May 24;25(10):2637–48. doi: 10.1007/s11605-021-05036-8 (PMC8523499; doi:10.1007/s11605-021-05036-8)
Supplement: Supplementary file 1 — (DOCX 24 kb). [file 11605_2021_5036_MOESM1_ESM.docx]

**SUPPLEMENTARY TABLE 1: Multilevel analyses for pathologic outcomes**

|  |  | **Incomplete resection margin  T1-3 colon carcinoma** | **Incomplete resection margin T1-3 rectal carcinoma** | **Incomplete resection margin T4 colon carcinoma** | **Incomplete resection margin   T4 rectal carcinoma** |
| --- | --- | --- | --- | --- | --- |
|  |  | Adj. OR  (95% CI) | Adj. OR  (95% CI) | Adj. OR  (95% CI) | Adj. OR  (95% CI) |
| **Tumor complications** | No | 1.00 (ref.) | 1.00 (ref.) | 1.00 (ref.) | 1.00 (ref.) |
|  | Yes | 1.043 (0.842-1.291) | 1.192  (1.024-1.388)* | 1.031 (0.879-1.210) | 0.968  (0.762-1.229) |
| **Neoadj. radiotherapy** | No | - | 1.00 (ref.) | - | 1.00 (ref.) |
|  | Yes | - | 1.310  (1.136-1.510)* | - | 1.080  (0.782-1.494) |
| **Setting** | Elective | 1.00 (ref.) | 1.00 (ref.) | 1.00 (ref.) | 1.00 (ref.) |
|  | Emergency | 1.686 (1.316-2.161)* | 2.925  (1.975-4.330)* | 1.680  (1.430-1.973)* | 2.108  (1.145-3.881)* |
| **Add. resection for local ingrowth** | No | 1.00 (ref.) | 1.00 (ref.) | 1.00 (ref.) | 1.00 (ref.) |
|  | Yes | 2.633 (2.039-3.402)* | 2.833  (2.292-3.500)* | 1.906 (1.662-2.187)* | 1.917  (1.522-2.414)* |
| **T-stage^A^** | T1-2 | 1.00 (ref.) | 1.00 (ref.) | - | - |
|  | T3 | 1.096 (0.748-1.606) | 1.238 (1.066-1.438)* | - | **-** |
| **N-stage** | N0 | 1.00 (ref.) | 1.00 (ref.) | 1.00 (ref.) | 1.00 (ref.) |
|  | N1 | 2.335 (1.852-2.944)* | 2.423  (2.109-2.783)* | 0.922 (0.770-1.104) | 1.532  (1.286-1.979)* |
|  | N2 | 4.823 (3.803-6.116)* | 4.540  (3.885-5.305)* | 1.492 (1.258-1.769)* | 2.422  (1777-3.300)* |
| **M-stage** | M- | 1.00 (ref.) | 1.00 (ref.) | 1.00 (ref.) | 1.00 (ref.) |
|  | M1 | 2.688 (2.169-3.332)* | 1.784  (1.506-2.114)* | 2.092 (1.810-2.418)* | 1.836  (1.418-2.377)* |
| **Tumor location** | Solitary | 1.00 (ref.) | 1.00 (ref.) | 1.00 (ref.) | 1.00 (ref.) |
|  | Synchronous | 3.848 (2.896-5.114)* | 0.847  (0.585-1.226) | 1.676 (1.238-2.269)* | 0.979  (0.417-2.296) |

Suppl. Table 1: Multilevel analyses for the primary pathologic endpoints of this study. A: For colon cancer, tumor stage is based on the pathologic tumor stage, whereas rectal cancer tumor stage is based on the clinical tumor stage. Neoadj. radiotherapy: neoadjuvant radiotherapy. Add. Resection for local ingrowth: additional resection for local ingrowth. Number of patients included per multilevel analyses are n=56,202 for incomplete resection margin T1-3 colon carcinoma, n = 11,211 for incomplete resection margin T4 colon carcinoma, n=22,705 for incomplete resection margin T1-3 rectal carcinoma, and n = 2,549 for incomplete resection. Statistical significant associations between variable and outcome are highlighted by*.

**SUPPLEMENTARY TABLE 2: Multilevel analyses for surgical outcomes**

|  |  | **Complicated course  colon cancer** | **Complicated course  rectal cancer** | **Failure to rescue  colorectal cancer** | **Mortality  colon cancer** | **Mortality  rectal cancer** |
| --- | --- | --- | --- | --- | --- | --- |
|  |  | Adj. OR  (95% CI) | Adj. OR  (95% CI) | Adj. OR  (95% CI) | Adj. OR  (95% CI) | Adj. OR  (95% CI) |
| **Age** | <60 | 1.00 (ref.) | 1.00 (ref.) | 1.00 (ref.) | 1.00 (ref.) | 1.00 (ref.) |
|  | 60-70 | 1.103  (1.026-1.186)* | 0.990  (0.911-1.076) | 2.226 (1.638-3.024)* | 1.860 (1.420-2.435)* | 2.478 (1.446-4.246)* |
|  | 70-80 | 1.258 (1.171-1.350)* | 1.048 (0.960-1.144) | 3.129 (2.326-4.209)* | 2.753 (2.127-3.563)* | 4.936 (2.947-8.267)* |
|  | 80+ | 1.668 (1.543-1.803)* | 1.196 (1.060-1.144)* | 7.390 (5.476-9.970)* | 6.466 (5.000-8.362)* | 9.945 (5.819-16.997)* |
| **Sex** | Male | 1.00 (ref.) | 1.00 (ref.) | 1.00 (ref.) | 1.00 (ref.) | 1.00 (ref.) |
|  | Female | 0.685 (0.655-0.717)* | 0.590 (0.551-0.632)* | 0.916 (0.806-1.041) | 0.727 (0.654-0.807)* | 0.547 (0.421-0.711)* |
| **BMI** | 18.5-25.0 | 1.00 (ref.) | 1.00 (ref.) | 1.00 (ref.) | 1.00 (ref.) | 1.00 (ref.) |
|  | <18.5 | 1.506  (1.304-1.739)* | 1.212 (0.947-1.552) | 1.487 (1.007-2.200)* | 2.235 (1.718-2.907)* | 1.204 (0.521-2.782) |
|  | 25.0-30.0 | 1.034 (0.984-1.087) | 1.000 (0.932-1.073) | 0.900 (0.786-1.031) | 1.027 (0.914-1.154) | 0.660 (0.510-0.855)* |
|  | >30 | 1.152 (1.083-1.226)* | 1.343 (1.230-1.466)* | 0.859 (0.728-1.015) | 1.093 (0.943-1.267) | 1.042 (0.767-1.415) |
| **ASA score** | I-II | 1.00 (ref.) | 1.00 (ref.) | 1.00 (ref.) | 1.00 (ref.) | 1.00 (ref.) |
|  | III+ | 1.704 (1.622-1.790)* | 1.452 (1.339-1.574)* | 2.282 (2.005-2.597)* | 3.179 (2.831-3.569)* | 2.632 (2.061-3.361)* |
| **CCI** | 0-I | 1.00 (ref.) | 1.00 (ref.) | 1.00 (ref.) | 1.00 (ref.) | 1.00 (ref.) |
|  | II+ | 1.331 (1.267-1.398)* | 1.128 (1.044-1.218)* | 1.412 (1.245-1.602)* | 1.653 (1.484-1.842)* | 1.461 (1.149-1.858)* |
| **Tumor complications** | No | 1.00 (ref.) | 1.00 (ref.) | 1.00 (ref.) | 1.00 (ref.) | 1.00 (ref.) |
|  | Yes | 1.143 (1.085-1.204)* | 1.139 (1.051-1.235)* | 1.038 (0.911-1.183) | 1.154 (1.023-1.303)* | 1.203 (0.926-1.563) |
| **Neoadj. radiotherapy** | No | - | 1.00 (ref.) | 1.00 (ref.) | - | 1.00 (ref.) |
|  | Yes | - | 1.242 (1.154-1.337)* | 0.856 (0.670-1.092) | - | 1.143 (0.890-1.986) |
| **Setting** | Elective | 1.00 (ref.) | 1.00 (ref.) | - | 1.00 (ref.) | 1.00 (ref.) |
| Emergency | | 1.876 (1.760-1.997)* | 1.787 (1.358-2.352)* | - | 2.459 (2.159-2.800)* | 3.022 (1.712-5.337)* |
| **Add. resection for local ingrowth** | No | 1.00 (ref.) | 1.00 (ref.) | 1.00 (ref.) | 1.00 (ref.) | 1.00 (ref.) |
|  | Yes | 1.538 (1.433-1.649)* | 1.477 (1.301-1.676)* | 0.862 (0.701-1.059) | 1.186 (1.009-1.394)* | 1.455 (0.949-2.232) |
| **Add. resection for metastases** | No | 1.00 (ref.) | 1.00 (ref.) | 1.00 (ref.) | 1.00 (ref.) | 1.00 (ref.) |
|  | Yes | 1.524 (1.359-1.709)* | 1.300 (1.073-1.576)* | 0.819 (0.591-1.136) | 1.206 (0.925-1.572) | 0.981 (0.485-1.985) |
| **T-stage^A^** | T1-2 | 1.00 (ref.) | 1.00 (ref.) | 1.00 (ref.) | 1.00 (ref.) | 1.00 (ref.) |
|  | T3 | 1.140 (1.077-1.206) | 1.012 (0.937-1.092) | 1.110  (0.964-1.278) | 1.137 (0.983-1.316) | 0.952 (0.729-1.244) |
|  | T4 | 1.388 (1.287-1.496)* | 1.058 (0.930-1.204) | 1.184 (0.959-1.462) | 1.526 (1.278-1.822)* | 0.873 (0.550-1.383) |
| **M-stage** | M- | 1.00 (ref.) | 1.00 (ref.) | 1.00 (ref.) | 1.00 (ref.) | 1.00 (ref.) |
|  | M1 | 1.049 (0.976-1.127) | 0.902 (0.797-1.020) | 1.652 (1.346-2.029)* | 1.620 (1.398-1.876)* | 1.447 (0.980-2.138) |
| **Tumor location** | Solitary | 1.00 (ref.) | 1.00 (ref.) | 1.00 (ref.) 0.680 (0.550-0.842)*^ | 1.00 (ref.) | 1.00 (ref.) |
|  | Synchronous | 1.796 (1.614-1.998)* | 1.265 (1.072-1.491)* | 0.978 (0.727-1.315)^^ 1.300 (0.826-2.044)^^^ | 1.361 (1.061-1.747)* | 1.924 (1.262-2.932)* |

Suppl. Table 2: Multilevel analyses for the surgical postoperative primary endpoints of this study. A tumor stage: complicated course and mortality after colon cancer resection is corrected for the pT-stage, whereas failure to rescue, complicated course and mortality after rectal cancer resection is corrected for the cT stage. Neoadj. radiotherapy: neoadjuvant radiotherapy. Add. resection for local ingrowth: additional resection for local ingrowth. Add. resection for metastases: additional resection for metastases. In failure to rescue the reference group of the number of tumors is the solitary colon tumor group, below the OR and CI for resp. solitary rectal tumors^, synchronous colon tumors^^, and synchronous rectal tumors^^^. Number of patients included per multilevel analyses are n=64,872 for complicated course after colon cancer resection, n = 24,940 for complicated course after rectal cancer resection, n =13,124 for failure to rescue, n = 64,872 for mortality after colon cancer resection, and n = 24,940 for mortality after rectal cancer resection. Statistical significant associations between variable and outcome are highlighted by*.
